# Cystic lymphangioma in a 44-year-old woman

**DOI:** 10.11604/pamj.2017.26.33.11579

**Published:** 2017-01-23

**Authors:** Moncef Sellami, Abdelmonem Ghorbel

**Affiliations:** 1 Department of Otorhinolaryngology-Head and Neck Surgery, Habib Bourguiba University Hospital, Sfax, Tunisia

**Keywords:** Cystic lymphangioma, head and neck, surgical treatment

## Image in medicine

Cystic lymphangioma is a congenital malformation of the lymphatic system. This condition is usually observed among children and very rarely among adults. The aetiology in adults is likely due to delayed proliferation of cell rests due to physical injury or infection. Clinically, cystic lymphangioma is asymptomatic in adults. Treatment is generally surgical, although other methods, such as aspiration, radiation and sclerotherapy have been used. We report a case of a 44-year-old woman presented with one-year history of slowly enlarging cervical swelling at the right side of the neck. The patient had no medical problems or history of trauma to the neck. Examination showed a 4 cm smooth, soft swelling of right supraclavicular fossa. Moreover, there was no erythema or tenderness on palpation. The general examination revealed normal findings. Computed tomography (CT) showed a supraclavicular cyst-like structure of 5×3×3 cm. The patient was operated through an external approach and a complete cyst excision was achieved without complications. Histopathological examination was consistent with the diagnosis of cystic lymphangioma. The patient made an uneventful recovery with no recurrence noted one year after the surgery.

**Figure 1:**
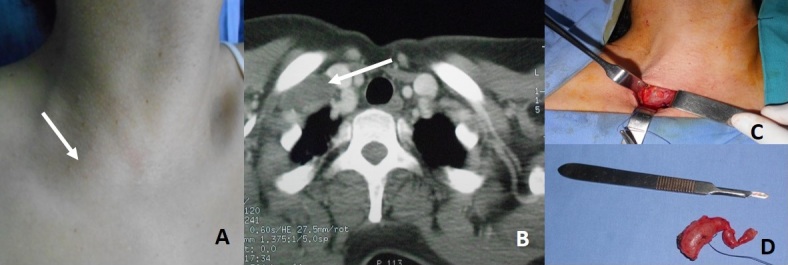
A) right supraclavicular mass on examination (arrow); (B) CT scan of the neck, showing a well-defined supraclavicular cystic mass (arrow); (C) Intra-operatively the mass appeared fluid filled and lobulated; (D) Excision of the cystic mass

